# SNP-Based Chromosomal Microarray Analysis in the Era of Optical Genome Mapping: An Enriched Case-Series Evaluating Copy-Neutral Events

**DOI:** 10.3390/cancers18111841

**Published:** 2026-06-04

**Authors:** Alexander R. Marr, Patrick R. Gonzales, Shivani Golem

**Affiliations:** Department of Pathology and Laboratory Medicine, University of Kansas Medical Center, Kansas City, KS 66160, USA; amarr@kumc.edu (A.R.M.); pgonzales@kumc.edu (P.R.G.)

**Keywords:** cytogenetics, optical genome mapping, chromosomal microarray, copy-neutral loss of heterozygosity, karyotype, genomics

## Abstract

Background/Objectives: Chromosomal microarray analysis (CMA) is an essential tool in modern cytogenetics for detecting copy number alterations and copy-neutral loss of heterozygosity (CN-LOH). As optical genome mapping (OGM) emerges as a potential replacement for traditional cytogenetic methods, the extent to which CMA remains necessary in routine diagnostic workflows remains to be elucidated. Methods: We retrospectively reviewed 53 primary neoplastic cases, selected from a larger cohort of 327 hematologic malignancy specimens, in which CMA identified one or more CN-LOH events. Event size, genomic content, and correlation with next-generation sequencing (NGS) findings were assessed. A separate cohort of newly diagnosed B-cell acute lymphoblastic leukemia (B-ALL) was analyzed to evaluate disease-specific CN-LOH frequency. Results: Nearly half of CN-LOH events detected were <25 Mb, below the current detection threshold of OGM inferred from published benchmarks and validated workflows. Many encompassed clinically relevant genes, including *FLT3*, *JAK2*, *TET2*, *TP53*, and *RUNX1*. Additionally, two-thirds of cases harbored pathogenic or likely pathogenic variants by NGS within the corresponding CN-LOH regions, further underscoring the clinical value of detecting these copy-neutral events. In contrast, CN-LOH was uncommon in B-ALL, and most alterations identified by CMA would be detectable by OGM. Many of these patients also harbored complex structural rearrangements that required multiple conventional assays for full characterization; these could be resolved by OGM in a single analysis. Conclusions: Our findings indicate that although OGM excels at resolving complex structural variants, CMA remains essential for detecting copy-neutral events. Until OGM achieves improved sensitivity for CN-LOH, an integrated approach utilizing conventional cytogenetics, CMA, NGS, and OGM provides the most reliable framework for comprehensive genomic assessment across cancer types.

## Simple Summary

Cytogenetic testing plays a key role in diagnosis and treatment selection for many blood cancers. Several laboratory methods are currently used, each with different strengths and limitations. A new technology called optical genome mapping has the potential to replace older methods by providing a comprehensive and streamlined view of complex genetic changes. However, it may miss smaller but clinically important alterations that can affect treatment decisions. In our study, we reviewed patient cases performed at our institution to determine how frequently these small changes occur and whether they remain clinically important. We found many of these alterations to involve key cancer genes and often occur alongside other mutations that influence disease behavior. Our findings suggest that combining multiple testing methods provides the most complete and reliable genetic assessment for cancer patients until technology improves.

## 1. Introduction

Classical cytogenetics has served as the cornerstone for diagnostic and prognostic classification of cancers for decades but remains limited by relatively low-resolution technologies and high labor intensity [1,2,3]. Identification of hallmark chromosomal abnormalities is integral for clinical diagnosis, risk stratification, and therapeutic decision-making, particularly in hematologic malignancies [3,4]. Technological advances such as chromosomal microarray (CMA) have broadened the scope of cytogenetic testing, enabling genome-wide detection of copy number (CN) variants and copy-neutral events, such as copy-neutral loss-of-heterozygosity (CN-LOH) [5,6]. Several clinically significant regions of CN-LOH have been identified on 4q, 9p, 13q, and 17p, among others, and their accurate recognition has become essential for routine testing and prognostic stratification [5,7].

Current gold-standard practice in cytogenetics laboratories relies on a combination of conventional karyotyping and fluorescence in situ hybridization (FISH) [1,3]. Identification of recurrent abnormalities, such as deletions of 5q and 7q in acute myeloid leukemia (AML), provides important prognostic information to guide treatment selection [4]. However, similar to all currently available cytogenetic methodologies, these techniques have technical limitations. For example, FISH is limited to predefined genomic regions, while karyotyping is technically challenging, time-consuming, and constrained by low-resolution capabilities [7,8,9]. These challenges have driven the need for high-resolution, genome-wide solutions.

CMA offers a genome-wide, high-resolution capability for detecting submicroscopic CN changes and regions of allelic imbalance [6]. Importantly, SNP-based CMA platforms can also detect small CN-LOH events within clinically significant regions, aiding prognostic stratification [10]. Currently, CMA has become an established complementary method to conventional karyotyping and FISH for both neoplastic and constitutional testing [3,6,10]. However, its inability to detect balanced rearrangements necessitates the use of additional molecular methods.

The limitations of current cytogenetic techniques have created demand for comprehensive, streamlined technology. Optical Genome Mapping (OGM) has emerged as such a platform, offering direct, high-resolution imaging of ultra-high-molecular-weight DNA to visualize structural variants [11,12]. Often described as “next-generation cytogenetics,” OGM is increasingly adopted by clinical laboratories for the characterization of structural variants in cancer [11,12,13,14,15]. Multiple studies have highlighted OGM’s key strengths, including excellent breakpoint resolution and high sensitivity for variants >500 bp in size [16,17,18]. Further, cases with complex karyotypes, defined as ≥3 clonal abnormalities [19,20], can be easily resolved, and workflows are streamlined compared to conventional karyotyping.

However, OGM also has limitations, including reduced detection performance in repetitive genomic regions, limited sensitivity for low-frequency variants, and the requirement for high-molecular-weight DNA, which can be technically challenging to achieve [21]. Additionally, the initial cost of instrumentation may limit the adoption of OGM at low-resource centers [21]. For these reasons, conventional cytogenetic approaches remain relevant and are still routinely used in diagnostic workflows. Despite these limitations, the strengths of OGM position it as an attractive alternative or complementary technology to traditional cytogenetic techniques, with the potential to consolidate multiple workflows into a single assay.

Despite its potential advantages, OGM’s sensitivity for CN-LOH detection is a key limitation and could be particularly problematic in disorders where biallelic inactivation is significant [15,22]. For example, homozygosity for driver mutations in genes such as *JAK2*, *CBL*, *TET2*, or *TP53* can be essential findings for clinical management, particularly when NGS detects pathogenic or likely pathogenic variants within the affected region [23,24,25,26]. While OGM excels at characterization of structural rearrangements, its lack of allelic resolution represents a critical diagnostic and prognostic constraint. Accurate detection of CN-LOH remains vital for comprehensive genomic interpretation and optimal clinical management in the age of precision medicine.

As laboratories transition to modern genomic technologies, the question remains whether CMA should still have a place in current workflows. To address this, we conducted a retrospective, enriched case-series analysis of 53 neoplastic CMA cases from the University of Kansas Health System (TUKHS) that included at least one CN-LOH event. Our findings demonstrate that, although OGM would enhance structural-variant detection, clinically significant focal copy-neutral events may go undetected. These results highlight the continued importance of performing CMA as a complementary modality in the ascendant era of OGM.

## 2. Materials and Methods

### 2.1. Patient Samples

This study was designed as a retrospective, enriched case-series analysis focusing on CN-LOH-positive cases identified within a larger clinical CMA cohort. CMA was performed on 327 patients with hematological malignancies at TUKHS in 2025. Within this cohort, a subset of 53 patients with CN-LOH events was then selected for full retrospective analysis. The cancer diagnosis was performed on blood or bone marrow by morphological and flow cytometry evaluations, as a standard clinical practice. Next-generation sequencing (NGS) using the QIAseq Targeted DNA Human Myeloid Neoplasms 141 gene Panel (Qiagen, Germantown, MD, USA) was also performed. Clinical significance of NGS variants was reported as per published guidelines [27]. The results of these evaluations were retrospectively reviewed from the patient’s medical records.

Karyotyping, FISH, and Neoplastic CMA were performed at TUKHS Cytogenetics Laboratory. No patients were excluded. All testing was performed as part of routine clinical care. The retrospective review was approved by an institutional review board (Study ID: STUDY00160638, approved on 20 June 2024).

### 2.2. Karyotyping and FISH Analysis

Conventional karyotyping and FISH analyses were performed on cultured peripheral blood or bone marrow specimens using standard cytogenetic protocols. For conventional karyotyping, a minimum of 20 metaphases were evaluated for each case. FISH testing included acute myeloid leukemia (AML) FISH probes *RUNX1T1*::*RUNX1*, *KMT2A*, *PML*::*RARA*, *MYC*, and 7q probes from Abbott (Des Plaines, IL, USA), and 5q, *CBFB*::*MYH11*, *MECOM*, and *PML*::*RARA* from Cytocell (Tarrytown, NY, USA) and *TP53* from MetaSystems (Medford, MA, USA). B-cell acute lymphoblastic leukemia (B-ALL) FISH probes included *BCR*::*ABL1*, *KMT2A*, *CDKN2A*, *IGH*, and *MYC* from Abbott (Des Plaines, IL, USA), *ETV6*::*RUNX1*, *ABL2*, *PDGFRB*, *JAK2*, *EPOR*, *CRLF2*, and *P2RY8* from Cytocell (Tarrytown, NY, USA), and *IKZF1* from Empire Genomics (Depew, NY, USA). 200 interphase cells were scored for FISH in all cases with new diagnoses. For follow-up cases, 500 interphase cells were scored. Metaphase FISH was not utilized in this analysis. Chromosome and FISH analyses were performed utilizing Cytovision Software version 7.7 (Leica, Teaneck, NJ, USA). Results were interpreted and reported using the International System for Human Cytogenomic Nomenclature (ISCN 2024).

### 2.3. DNA Extraction

Genomic DNA was extracted from peripheral blood or bone marrow using the QIA amp DNA Blood Mini Kit (Qiagen, Germantown, MD, USA) according to the manufacturer’s instructions. DNA was quantified by Qubit fluorometry. At least 200 ng of total DNA was utilized for CMA.

### 2.4. Chromosomal Microarray

Microarray-based chromosome analysis was performed using the iScan System with the Global Diversity Array-8 (GDACyto) v1.0 Array BeadChip (Illumina, San Diego, CA, USA). Criteria for designating reportable aberrations include gains or losses larger than 50 kb involving clinically significant cancer genes; gains >2 Mb; and losses >1 Mb outside known clinically significant oncology regions that span at least one annotated RefSeq gene. Smaller aberrations are reported only if the regions are likely to be clinically significant. Copy-neutral loss of heterozygosity (CN-LOH) is reported when the region exceeds 3 Mb. CMA and visualization were performed using NxClinical 6.2 (Bionano, San Diego, CA, USA).

All NGS and CMA findings are annotated using the human genome reference build GRCh37/hg19.

### 2.5. Statistics

Descriptive statistics, including frequencies, median values, and distribution counts, were generated in Microsoft Excel or R. Figures were generated using R (v4.5.1).

## 3. Results

### 3.1. Assessing the Frequency and Impact of CN-LOH Events in Routine Neoplastic Testing

TUKHS Cytogenetics Laboratory performed neoplastic CMA on 327 patients aged 18 to 92 years in 2025. 184 patients were male, 142 were female, and one identified as other. Within this cohort, 53 patients were selected for a retrospective, enriched case series analysis to evaluate the prospective clinical value that OGM could have provided in lieu of CMA. The average patient age of the selected cohort was 64 years, with 28 male and 25 female cases. The full demographic, cytogenetic, and molecular results of our patient cohort are summarized in Supplementary Table S1. These cases served as the foundation of our comparative analysis of CMA and potential OGM coverage.

**Figure 1:**
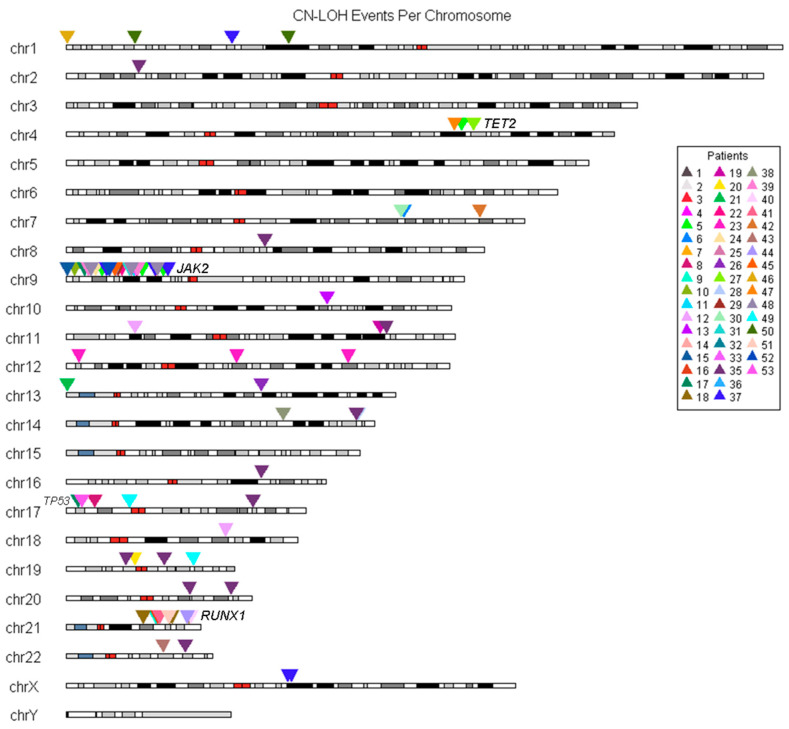
Idiograms of chromosomes 1-22, X, and Y are displayed with CN-LOH events plotted as inverted triangles positioned at approximate genomic locations. Individual cases are distinguished by unique colors. Chromosomes without markers indicate absence of CN-LOH calls in that region. Overlapping CN-LOH events are horizontally offset by 500 kb to improve visibility.

**Figure 2:**
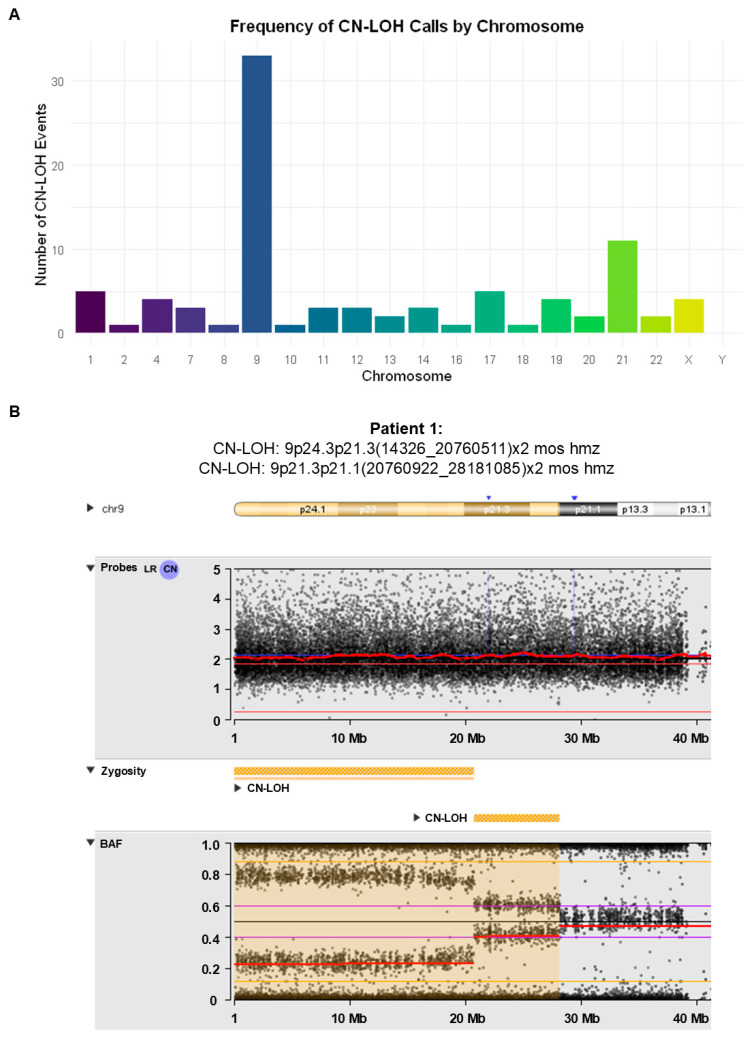
(**A**) Histogram depicting the quantity of CN-LOH events by chromosome number (*n* = 85 events total). (**B**) Patient 1: CMA of chromosome 9p shows diploid copy number (CN probe median ~2; red smoothed line) without copy number alterations. The B-allele frequency (BAF; purple heterozygous imbalance thresholds) shows clustering of probes away from the 0.5 heterozygous band across two contiguous segments on 9p. Integration of CN and BAF profiles indicates mosaic CN-LOH, which is supported by the zygosity track (gold bars). Corresponding ISCN nomenclature for the two events is indicated above the illustration.

A genome-wide map of CN-LOH events from our selected cohort is shown in Figure 1. A total of 85 CN-LOH calls were detected (median ~2 per case), ranging from focal (<1 Mb) to whole arm (>110 Mb) in size. 42 CN-LOH calls (49% of total) were <25 Mb in length, a common benchmark identified by multiple previous studies, where OGM would likely not detect these calls [14,22,28]. The distribution of CN-LOH calls by chromosome number in our cohort is highlighted in Figure 2A. The most common CN-LOH event detected involved the *JAK2* gene on 9p, observed in 37% of all cases evaluated. Frequently, multiple adjacent regions of CN-LOH on 9p are identified, such as in patient 1 (Figure 2B).

**Figure 3:**
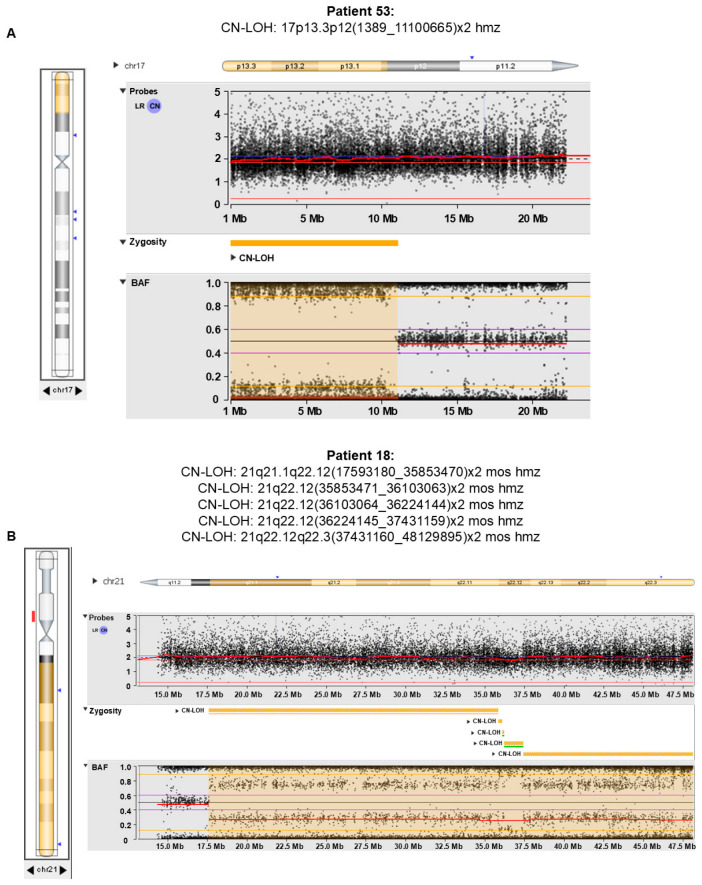
(**A**) Patient 53; (**B**) Patient 18. CMA of chromosome 17p (*TP53*) and chromosome 21q (*RUNX1*), respectively, shows a diploid copy number (CN probe median of ~2; red smoothed line) without copy number alterations. The B-allele frequency (BAF; purple heterozygous imbalance thresholds) shows clustering of probes away from the 0.5 heterozygous band across segments on 17p and 21q, respectively. Integration of CN and BAF profiles indicates mosaic CN-LOH, supported by the zygosity track (gold bars). Corresponding ISCN nomenclature is indicated above the illustration.

We further investigated the relationship of CN-LOH, karyotype, and FISH results by primary diagnosis, as shown in Table 1. 35 of 53 cases (66%) with CN-LOH detected had a normal karyotype, with the diagnosis of MPN having the highest percentage of cases with normal karyotype occurring in 76%, followed by AML (61%), and MDS (60%). By contrast, six cases showed complex structural abnormalities (≥3) on karyotype. In these cases, OGM would have offered significant advantages for resolving these complex rearrangements while still identifying the large CN-LOH regions. However, CN-LOH events identified by CMA in four of these individuals were <25 Mb in length. For example, in patient 8 (MDS) and patient 53 (AML), a 17p CN-LOH was identified of approximately 20 and 11 Mb in size, respectively, which included the tumor suppressor gene *TP53* (17p13.1) (Figure 3A). *TP53* p.Y234D and p.Y236C NGS variants were also present at 56% and 86% variant allele fraction (VAF), respectively. These findings are consistent with biallelic inactivation of the *TP53* gene.

Further analysis revealed that 41 of the 53 patients (77.4%) harbored clinically actionable NGS-identified variants within the CN-LOH regions. Genes in these regions included *FLT3* (13q12.2) (seen in 2/53 (3.7%) total; 2/14 (14.2%) AML), *JAK2* (9p24.1) (20/53 (37.7%) total; 2/14 (14.2%) AML; 1/15 (6.6%) MDS; 16/21 (76.1%) MPN, one case of CMML), *TET2* (4q24) (4/53 (7.5%) total; 2/15 (13.3%) MDS; 1/21 (4.7%) MPN; one case of CMML), *RUNX1* (21q22.12) (5/53 (9.4%) total; 4/14 (28.6%) AML; one case of CML), *MPL* (1p34.2) (2/53 (3.7%) total; 2/21 (9.5%) MPN), *TP53* (17p13.1) (2/53 (3.7%) total; one case of MDS, one case of AML), *EZH2* (7q36.1) (seen in 2/53 (3.7%) total; 2/15 (13.3%) MDS), *NOTCH1* (9q34.3) (patient 15 with T-ALL), *CDKN2A* (9p21.3) (patient 31 with B-ALL), *BCOR* (Xp11.4) (patient 34 with MDS), *SETBP1* (18q12.3) (patient 12 with MDS/MPN), *U2AF1* (21q22.3) (patient 40 with MDS). Although *TET2* single- and double-hit mutations were detected in 4/14 (28.6%) AML cases by NGS, CN-LOH was not present. CN-LOH of 21q involving *RUNX1* mutation, the sole abnormality detected in AML cases, was associated with a normal or intermediate-risk karyotype; an example is shown for patient 18 (Figure 3B). These findings highlight the complementary value of integrating CMA with NGS, as CN-LOH can augment the impact of existing variants. For example, identification of *TP53* biallelic status is critical for recommendations for hematopoietic cell transplantation or clinical trial enrollment [29].

### 3.2. Newly Diagnosed B-ALL Cases

To better illustrate OGM’s potential advantages in a cytogenetically complex disease context, we identified 14 patients with newly diagnosed B-ALL who underwent concurrent karyotyping, FISH, and CMA within our total CMA testing cohort. This subgroup was selected as a representative example of a disease entity characterized by frequent structural rearrangements. At our institution, all newly diagnosed B-ALL cases undergo CMA as part of routine clinical workup, enabling comprehensive cytogenetic characterization. The full clinical results of our B-ALL cohort are summarized in Table 2. The average patient age for this cohort was 53 years, with 11 male and 3 female cases.

Most of our B-ALL cohort demonstrated both abnormal karyotype and FISH results. Our laboratory routinely performs *BCR::ABL1* “STAT” FISH testing on peripheral blood, followed by a cascade of additional probes, including *KMT2A*, *IKZF1*, *CDKN2A*, *ETV6::RUNX1*, and *IGH*. Cases reported positive for *BCR::ABL1* reflex to a Ph-like panel, including *ABL2*, *PDGFRB*, *JAK2*, *EPOR*, and *MYC* FISH probes. Further, *CRLF2*-positive immunophenotypes identified by flow cytometry trigger *CRLF2* and *P2RY8* FISH confirmatory testing. Collectively, this workflow often results in numerous FISH probes per case, even when many assays yield normal results. For example, patient 13 required 11 separate FISH assays, 10 of which were normal, as shown in Table 2. While some laboratories have adopted RT-PCR testing for common fusions followed by complementary FISH testing, our approach of comprehensive FISH testing at diagnosis is aligned with NCCN guidelines for detecting the major recurrent genomic abnormalities [30].

CN-LOH was identified in only one patient (patient 31; Supplementary Table S1) and was >25 Mb in length. However, focal copy-number changes <25 Mb were identified in 7 of the 13 successful CMA studies. Of note, one patient had canceled CMA because a low-hypodiploid karyotype was detected, with high prognostic risk.

## 4. Discussion

Our retrospective study highlights CN-LOH as a frequent and clinically significant finding in routine neoplastic testing. In our selected cohort of patients with CN-LOH events detected, nearly half of the CN-LOH events observed were <25 Mb in size and below the conventional OGM reporting threshold reported in recent studies [31]. Despite their smaller sizes, they encompass mutations in critical driver genes with direct implications for risk stratification and treatment selection, particularly when accompanied by NGS variants within the CN-LOH region. CMA offers distinct advantages derived from its single-nucleotide polymorphism (SNP)- based array design. The platform provides genome-wide coverage, enabling the detection of submicroscopic CN changes and copy-neutral events such as CN-LOH, uniparental disomy (UPD), and regions of homozygosity [6]. For example, CN-LOH involving the *TP53* gene, along with a clinically actionable *TP53* variant identified by NGS, has significant prognostic value in patients with MDS and AML. The resulting biallelic inactivation of *TP53* in MDS (patient 8) meets the criteria for the MDS-defining genetic subtype, MDS with biallelic *TP53* inactivation (MDS-bi*TP53*), per the World Health Organization (WHO) and National Comprehensive Cancer Network (NCCN.org), and is associated with increased bone marrow blasts and a high risk of progression to leukemia and death, independent of treatment [32,33]. In AML with biallelic *TP53* mutations (patient 53), individuals fall into a high-risk subset within the poor prognostic category of recurrent genetic abnormalities. Accurate determination of *TP53* biallelic status is increasingly critical for guiding precision medicine decisions, including recommendations for hematopoietic cell transplantation or clinical trial enrollment [29]. Detecting focal CN-LOH events involving the *TP53* locus provides essential insight into biallelic involvement, information that would be missed by OGM alone.

In our study, several 9p CN-LOH events co-occurred with *JAK2* mutations identified by NGS, a pattern associated with increased mutation burden, clonal expansion, and a higher risk of progression to myelofibrosis or accelerated phase, underscoring the importance of detecting these copy-neutral events [34,35]. Two AML patients harbored FLT3 internal tandem duplications with CN-LOH spanning the *FLT3* locus; prior work has linked this combination to higher relapse rates and poorer overall survival [36]. Additionally, *MPL* W515 mutations accompanied by CN-LOH involving the *MPL* locus were observed in patients with myelofibrosis. Acquired 1p CN-LOH affecting *MPL* has been implicated in fibrotic transformation in MPNs carrying *MPL* mutations [37].

CN-LOH involving 4q, encompassing *TET2*, was also frequently observed. *TET2* mutations are common in MDS, AML, and clonal hematopoiesis, and are associated with worse prognosis in MDS [38]. *TET2* inactivation through CN-LOH has been linked to increased proliferation, inflammation, and self-renewal [39]. Although not a distinct disease subtype, patients with high-VAF *TET2* mutations and CN-LOH carry a high risk of progressing from early leukemic states, such as clonal hematopoiesis, to overt malignancy. Early identification of these high-risk *TET2* events may enable earlier intervention and risk-reduction strategies. CN-LOH of 21q is another recurrent abnormality in AML [36,40]. In our cohort, 21q CN-LOH co-occurring with a pathogenic *RUNX1* variant was detected in 28% of AML cases. While one prior study reported favorable outcomes in AML with 21q CN-LOH, *RUNX1* mutation status was not evaluated [41]. In contrast, biallelic *RUNX1* alteration resulting from CN-LOH combined with a *RUNX1* sequence variant has been associated with a worse prognosis compared with monoallelic mutation [36,42]. These findings underscore the importance of integrating CMA and NGS for accurate clinical interpretation and highlight CMA’s high-resolution CN-LOH detection in guiding clinical decision-making.

OGM provides superior structural variant detection, high-resolution breakpoint mapping, and the ability to consolidate multiple cytogenetic workflows into a single assay [12,14,18]. These advantages are especially valuable in cases with complex rearrangements that are difficult to resolve by conventional karyotyping. However, practical considerations, including institutional resources and technical requirements, must be addressed when developing an OGM-based workflow. Implementation requires a significant upfront investment, including ultra-high-molecular-weight DNA extraction, specialized instrumentation, and substantial computational infrastructure, which may be challenging for smaller laboratories with limited budgets or support. Although these barriers are expected to diminish as technology matures, they currently remain a constraint for many centers.

Across myeloid malignancies, including MPN, MDS, and AML, 60–76% of cases showed normal karyotypes with CN-LOH identified only by CMA (Table 1). Recent data indicate limited clinical utility of OGM in MPN: aside from detecting *KMT2A*-PTD in a subset of karyotypically normal cases, OGM did not reveal additional tier 1 or tier 2 copy-number or structural variants in MDS or MDS/MPN, nor did it identify any new tier 1 abnormalities in myeloid malignancies with complex karyotypes. In AML, tier 2 OGM-detected abnormalities mainly involved *MECOM*, *KMT2A*, and *NUP98* rearrangements, [43] most of which would be captured by CMA and the AML FISH panel used in this study for adult and elderly patients; *NUP98* FISH may be useful in pediatric settings. Based on these findings, we propose that laboratories without OGM perform karyotyping, a heme NGS panel, and CMA for MPN, MDS, and MDS/MPN, with reflex FISH testing based on karyotype findings for future minimal residual disease detection. For adult AML, we suggest karyotyping, targeted FISH for actionable rearrangements requiring rapid turnaround, CMA, and a heme NGS panel. For laboratories implementing OGM, testing should include limited FISH for urgent, actionable rearrangements; CMA until segmental or focal CN-LOH detection is available in OGM; a heme NGS panel; and OGM.

Newly diagnosed B-ALL requires extensive cytogenetic evaluation, often using multiple targeted FISH probes that are costly, labor-intensive, and restricted to predefined loci. OGM offers a comprehensive, streamlined alternative. Although multiple studies suggest it cannot detect focal CN-LOH, these events are less common in B-ALL than in myeloid neoplasms [31,44]. In contrast, the structural variants, aneuploidies, fusions, and rearrangements that characterize B-ALL are strengths of OGM detection. In our cohort, OGM would have identified all clinically relevant abnormalities targeted by FISH in a single assay, reducing cost, labor, and turnaround time. Many focal copy-number alterations detected by CMA, such as *CDKN2A*, *RB1*, and *IKZF1* losses, also fall within OGM’s resolution for copy-number gains and losses (~500 bp), and OGM would concurrently detect structural rearrangements missed by CMA. We propose that diagnostic workflows for B- and T-ALL include karyotype, FISH, a heme NGS panel, and CMA for laboratories without OGM. For laboratories with OGM, we propose limiting FISH to rapidly actionable rearrangements while incorporating OGM and a heme NGS panel.

Although whole-genome sequencing (WGS) provides the most comprehensive assay for detecting CN changes, SVs, and CN-LOH, its clinical adoption is limited by the substantial sequencing depth, computational resources, and bioinformatics expertise required. Similar challenges apply to combined NGS-plus-SNP approaches and whole-exome sequencing (WES) [45]. WES can infer CN-LOH using BAF and read-depth modeling, but sensitivity is far lower than with WGS or SNP arrays due to limited exonic coverage. Accordingly, WGS, WES, and hybrid NGS-plus-SNP workflows were not included in the proposed testing recommendations for routine clinical use.

A future consideration in modern cytogenetics and genomic testing is the expanding role of artificial intelligence (AI) [46,47]. The paradigm of chromosome analysis is rapidly evolving with the integration of AI algorithms into karyotyping software [48]. Recent studies have demonstrated that AI-assisted karyotyping can improve the accuracy of detecting recurrent chromosomal abnormalities while significantly reducing analysis time, without compromising diagnostic transparency [48,49,50,51]. However, AI has not yet been widely implemented across other cytogenomic platforms. Continued development and integration of AI-driven analytical tools are expected to further streamline, scale, and improve the efficiency of diagnostic workflows.

The limitations of this study include its retrospective design and modest cohort size, which constrain generalizability. Additionally, OGM has not been clinically implemented at our institution due to resource limitations; therefore, parallel OGM analysis on our patient cohort was not performed, limiting the generalizability of our findings. Furthermore, no high-molecular-weight DNA was available retrospectively, precluding subsequent OGM analysis. Incorporating OGM in future analyses would significantly strengthen our results. However, prior reports indicating limited OGM sensitivity for focal CN-LOH confirm that many such events in our cohort would likely remain undetected [31]. Parallel SNP-based CMA and OGM comparison studies have demonstrated substantial variability in OGM-based CN-LOH detection based on analytical pipelines, with concordance rates ranging from 37% to 90% [21,31]. Importantly, the same size threshold of 25 Mb was determined for reduced detection sensitivity, further supporting our results [31]. Specifically, the authors state that 47% of the events detected by SNP-based CMA were <25 Mb and included clinically significant regions, consistent with other previous studies and with our results [31,52]. Our findings should not be interpreted as a head-to-head comparison, but rather as an evaluation of CN-LOH detection within a clinically annotated cohort to inform future cytogenetic workflows. Larger, multi-institutional studies across diverse hematologic malignancies, integrating karyotyping, FISH, CMA, OGM, and NGS, are needed to better define the complementary roles of these technologies and guide test selection based on available resources.

## 5. Conclusions

While OGM represents a significant advance in cytogenetic technology, our workflow-oriented and hypothesis-generating analysis demonstrates that CMA remains an essential diagnostic resource for detecting clinically relevant CN-LOH events. Rather than replacement, we suggest that the future of cytogenetic testing lies in strategic integration, in which the strengths of multiple technologies are leveraged to deliver a complete and actionable genetic profile. This combined approach ensures that no significant event is overlooked in the pursuit of precision oncology.

## Figures and Tables

**Table 1:** CN-LOH relationships to karyotype and FISH results by primary diagnosis.

Diagnosis	Total Cases	Karyotype Category			FISH Result			CMA Result	
		<3 Abnormalities	≥3 Abnormalities	Normal	Positive	Negative	Not Performed	LOH with CN Loss/Gain	LOH Without CN Loss/Gain
AML	14 (26%)	3	3	8	3	9	2	6	8
MDS	15 (28%)	5	1	9	1	0	14	8	7
CMML	1 (2%)	0	0	1	1	0	0	0	1
B-cell ALL	1 (2%)	0	1	0	1	0	0	1	0
T-ALL	1 (2%)	0	0	1	1	0	0	1	0
CML	1 (2%)	0	1	0	1	0	0	1	0
Essential Thrombocytopenia (ET)	2 (4%)	0	0	2	0	0	2	0	2
MPN	4 (8%)	1	0	3	0	0	4	0	4
Myelofibrosis (MF)	8 (15%)	2	0	6	1	0	7	2	6
Polycythemia Vera (PV)	6 (11%)	0	1	5	0	0	6	2	4
MPN (CML, ET, MPN, MF, PV)	21 (40%)	3	2	16	2	0	19	5	16

**Table 2:** Newly diagnosed B-ALL cohort demographic, cytogenetic, and molecular characteristics.

Patient	Age	Sex	Diagnosis	CMA Events	Size of LOH(s)	Karyotype	FISH	NGS (Variant Allele Fraction [VAF %])
1	72	F	B-cell ALL	CN Loss: 6q23.3(135360827_135437106)x1~2 CN Gain: 9q34.12q34.3(133736858_141127851)x2~3 CN Gain: 10p15.3p11.1(60523_39154220)x2~3 CN Gain: 10q11.21q26.3(42355302_135499683)x2~3 CN Gain: 22q11.1q11.23(16052962_23629737)x2~3	0.08 Mb 7.4 Mb 39.1 Mb 93.1 Mb 7.6 Mb	46,XX,t(9;22)(q34;q11.2)[15]/47,sl,+der(22)t(9;22)[2]/48,sdl,+10[3]	Bone Marrow: nuc ish (ABL1,BCR)x3(ABL1 con BCRx2)[166/200]/(ABL1,BCR)x4(ABL1 con BCRx3)[4/200],(IKZF1,7q11.21)x2[200]	RUNX1 p.R166Q (VAF: 47%)
2	57	F	B-cell ALL	CN Loss: (7)x1~2	159.1 Mb	45,XX,−7,t(9;22)(q34;q11.2)[12]/46,XX[8]	Peripheral Blood: nuc ish (ABL1,BCR)x3(ABL1 con BCRx2)[83/200],(IKZF1,7q11.21)x1[80/200],(CDKN2A,CEP9)x2[200]	Not performed
3	49	M	B-cell ALL	CN Loss: 7p12.2(50417522_50462935)x1~2	0.05 Mb	46,XY,t(9;22)(q34;q11.2)[20]	Peripheral Blood: nuc ish(ABL1,BCR)x3(ABL1 con BCRx2)[175/200],(IKZF1,7q11.21)x2[200]	Not performed
4	60	M	B-cell ALL	CN Loss: 7p22.3p14.1(16487_37534044)x1~2 CN Loss: 7p14.1p12.1(39905801_53103888)x1~2 CN Gain: (8)x2~3 CN Loss: 9p24.3p21.3(14326_21753137)x1~2 Homozygous Copy Loss: 9p21.3(21753138_21984661)x0~1 CN Loss: 9p21.3(21984662_38792812)x1~2 CN Loss: 15q13.2q13.3(30915983_32795582)x1 CN Loss: Xp22.33(60425_2698170)x1~2 High Copy Gain: Xq25(123154718_123237996)x3 CN Loss: Xq28(154936819_155236204)x1~2 Hemizygous Copy Loss: Yp11.31q12(2654333_28817636)x0~1	37.5 Mb 13.2 Mb 146.4 Mb 21.7 Mb 0.2 Mb 16.8 Mb 1.9 Mb 2.6 Mb 0.08 Mb 0.3 Mb 26.2 Mb	45,XY,−7,der(9)t(7;9)(q11.2;p13)[15]/46,XY[5]	Peripheral Blood: nuc ish (CDKN2Ax1,CEP9x2)[24/200],(IKZF1x1,7q11.21x2)[22/200],(ABL1,BCR)x2[200] Bone Marrow: nuc ish (ABL2x2)[200],(PDGFRBx2)[200],(MYCx2)[200],(JAK2x2)[200],(EPORx2)[200]	DDX41 c.465G>A p.M155I (VAF: 50%)
5	70	M	B-cell ALL	arr (X,Y)x1,(1-22)x2	N/A	46,XY,t(9;22)(q34;q11.2)[3]/46,XY[17]	Peripheral Blood: nuc ish (ABL1,BCR)x3(ABL1 con BCRx2)[115/200],(IKZF1,7q11.21)x2[200] Bone Marrow: nuc ish (ABL1,BCR)x3(ABL1 con BCRx2)[115/200],(IKZF1,7q11.21)[200],(CDKN2A,CEP9)x2[200]	Normal
6	63	M	B-cell ALL	Cancelled because low-hypodiploid karyotype was detected.	N/A	34~36,XY,−2,−3,−7,−9,−12,−13,−15,−16,−17,−20,−22[cp11]/46,XY[9]	Peripheral Blood: nuc ish (CDKN2A,CEP9)x1[118/200],(IKZF1,7q11.21)x1[108/200],(ABL1x1,BCRx2)[78/200]/(ABL1,BCR)x1[42/200/(ABL1x2,BCRx3)[16/200]/(ABL1x1,BCRx3)[5/200],(ETV6x1,RUNX1x2)[112/200],(KMT2Ax2)[200]	TP53 Q144* (VAF: 53%)
7	37	M	B-cell ALL	arr (X,Y)x1,(1-22)x2 (Low-level copy number alterations of 1–4, 6, 17, and 22 below reporting threshold)	N/A	46,XY[20]	Bone Marrow: nuc ish (ABL1x2,BCRx1)[135/200],(IKZF1,7q11.21)x2[200],(CDKN2A,CEP9)[200],(KMT2Ax2)[200],(ETV6,RUNX1)x2[200]	TP53 p.R248G (VAF: 32%); RB1 p.L561Gfs*52 (VAF: 5.9%)
8	24	M	B-cell ALL	CN Gain: (5)x2~3 CN Gain: (8)x2~3 CN Gain: (10)x2~3 CN Gain: (21)x2~3	180.9 Mb 146.4 Mb 135.5 Mb 48.1 Mb	51,XY,+X,+5,+8,+10,−16,+der(19)t(1;19)(q23;p13.3),+21[2]/46,XY[18]	Bone Marrow: nuc ish (CRLF2x2)(3’CRLF2 sep 5’CRLF2x1)[5/200]/(CRLF2x3)(3’CRLF2 sep 5’CRLF2x1)[9/200],(ABL2x3)[19/200],(MYCx3)[19/200],(ETV6x2,RUNX1x3)[19/200],(P2RY8x3)[18/200],(PDGFRBx3)[16/200],(KMT2Ax3)[9/200],(IKZF1,7q11,21)x2[200],(ABL1,BCR)x2[200],(CDKN2A,CEP9)x2[200],(JAK2x2)[200],(EPORx2)[200]	NOTCH1 c.4031C?T p.T1344M (VAF: 49%); PML c.1710+685C>T (VAF: 48%)
9	54	M	B-cell ALL	CN Loss: 9p21.3p13.1(20621028_39097054)x1~2	18.5 Mb	46,XY,del(9)(p23p21)[10]/46,XY[10]	Bone Marrow: nuc ish (CDKN2Ax1,CEP9x2)[129/200],(EPORx2)(3’EPOR sep 5’EPORx1)[5/200],(ABL2x2)[200],(PDGFRBx2)[200],(IKZF1,7q11.21)x2[200],(MYCx2)[200],(ABL1,BCR)x2[200],(JAK2x2)[200],(KMT2Ax2)[200]	NF1 p.L549Cfs*7 (VAF: 37%); BCORL1 p.Q1714Lfs*28 (VAF: 63%); BCORL1 p.Q1714_A1715ins* (VAF: 63%)
10	79	M	B-cell ALL	arr (X,Y)x1,(1-22)x2	N/A	46,XY,t(8;22)(p11.2;q11.2)[5]/46,XY[15]	Bone Marrow: nuc ish (FGFR1,D8Z2)x2(5’FGFR1 sep 3’FGFR1x1) [310/500],(ABL1x2,BCRx3)[92/200],(MYCx2)[200],(IGH,BCL2)x2[500]	CDKN2A c.156_193+1del (VAF: 55%)
11	50	M	B-cell ALL	CN Loss: 3p21.31(47129903_47204518)x1~2 CN Loss: 3q13.2(112053956_112219535)x1~2 CN Loss: 7p12.2(50411178_50463667)x1 CN Loss: 9p21.3(19926411_21833882)x1~2 Homozygous Copy Loss: 9p21.3(21833883_2207358)x0~1 CN Loss: 9p21.3(22007359_22706144)x1~2 CN Loss: 12q21.33(92227078_92537956)x1~2 Homozygous Copy Loss: 13q14.2(48985639_49073897)x0~1)	0.07 Mb 0.17 Mb 0.05 Mb 1.9 Mb 0.17 Mb 0.7 Mb 0.31 Mb 0.09 Mb	46,XY,der(3)add(3)(p25)add(3)(q27)[13]/46,XY[7]	Peripheral Blood: nuc ish (CRLF2x2)(3’CRLF2 sep 5’CRLF2x1)[85/200],(ABL1,BCR)x2[200],(P2RY8x2)[200] Bone Marrow: nuc ish (BCL6x2)[200]	SRSF2 p.P95H (VAF: 11%)
12	74	F	B-cell ALL	CN Loss: (7)x1~2	159.1 Mb	45,XX,−7,t(9;22)(q34;q11.2)[3]/46,XX[17]	Peripheral Blood: nuc ish (ABL1,BCR)x3(ABL1 con BCRx2)[78/200],(IKZF1,7q11.21)x1[34/200],(CDKN2A,CEP9)x2[200],(KMT2Ax2)[200],(ETV6,RUNX1)x2[200]	JAK2 p.R683G (VAF: 32%); NRAS p.G12A (VAF: 35%)
13	24	M	B-cell ALL	CN Gain: 1q12q44(142544928_249212725)x2~3 CN Gain: (10)x2~3 CN Gain: (21)x2~3 CN Gain: (22)x2~3	106.7 Mb 135.5 Mb 48.1 Mb 51.3 Mb	49,XY,+1,+22,inc[1]/46,XY[19]	Bone Marrow: nuc ish (ABL1x2,BCRx3)[8/200],(SCFD2,LNX,PDGFRA/KIT)x2[200],(IKZF1,7q11.21)x2[200],(FGFR1,D8Z2)x2[200],(MYCx2)[200],(CDKN2A,CEP9)x2[200],(JAK2x2)[200],(KMT2Ax2)[200],(ETV6,RUNX1)x2[200],(FLT3x2)[200],(EPORx2)[200]	NBN p.S53Cfs*9 (VAF: 48%)
14	24	M	B-cell ALL	CN Gain: 1q12q44(142544928_249212725)x2~3 CN Gain: (10)x2~3 CN Gain: (21)x2~3 CN Gain: (22)x2~3	106.7 Mb 135.5 Mb 48.1 Mb 51.3 Mb	49,XY,+1,+22,inc[1]/46,XY[19]	Bone Marrow: nuc ish (ABL1x2,BCRx3)[8/200],(SCFD2,LNX,PDGFRA/KIT)x2[200],(IKZF1,7q11.21)x2[200],(FGFR1,D8Z2)x2[200],(MYCx2)[200],(CDKN2A,CEP9)x2[200],(JAK2x2)[200],(KMT2Ax2)[200],(ETV6,RUNX1)x2[200],(FLT3x2)[200],(EPORx2)[200]	CBLC c.355C>T p.R119* (VAF: 49%)

## Data Availability

The data that support the findings of this study are available on request from the corresponding author. The data are not publicly available due to privacy or ethical restrictions.

## Supplementary Materials

The following supporting information can be downloaded at: https://www.mdpi.com/article/10.3390/cancers18111841/s1, Table S1: Patient cohort demographic, cytogenetic, and molecular characteristics.

## Abbreviations

The following abbreviations are used in this manuscript:

AI	Artificial intelligence
AML	Acute myeloid leukemia
ALL	Acute lymphoblastic leukemia
B-ALL	B-cell acute lymphoblastic leukemia
CMA	Chromosomal microarray
CN	Copy number
CN-LOH	Copy-neutral loss of heterozygosity
CMML	Chronic myelomonocytic leukemia
FISH	Fluorescence in situ hybridization
MDS	Myelodysplastic syndrome
MPN	Myeloproliferative neoplasm
NGS	Next-generation sequencing
NCCN	National Comprehensive Cancer Network
OGM	Optical genome mapping
UPD	Uniparental disomy
VAF	Variant allele fraction
WHO	World Health Organization
